# Does market exclusivity hinder the development of Follow-on Orphan Medicinal Products in Europe?

**DOI:** 10.1186/1750-1172-6-59

**Published:** 2011-09-05

**Authors:** Anne EM Brabers, Ellen HM Moors, Sonja van Weely, Remco LA de Vrueh

**Affiliations:** 1 NIVEL, Netherlands Institute for Health Services Research, PO Box 1568, 3500 BN Utrecht, the Netherlands; 2 Innovation Studies, Copernicus Institute, Utrecht University, Heidelberglaan 2, 3584 CS Utrecht, the Netherlands; 3 Dutch Steering Committee on Orphan Drugs, Laan van Nieuw Oost Indië 334, 2593 CE Den Haag, the Netherlands

## Abstract

**Background:**

We determined whether the market exclusivity incentive of the European Orphan Drug Regulation results in a market monopoly or that absence of another Orphan Medicinal Product (OMP) for the same rare disorder, a so-called follow-on OMP, is a matter of time or market size. In the interest of rare disorder patients better understanding of the effect of the market exclusivity incentive on follow-on OMP development is warranted.

**Methods:**

First, the impact of various market-, product- and disease-related characteristics on follow-on OMP development in the EU was determined by comparing rare disorders with an approved OMP and at least one follow-on OMP (N = 26), with rare disorders with an approved OMP and no follow-on OMP (N = 18). Next, we determined whether manufacturers continued development of a follow-on OMP upon approval of the first OMP for the intended rare disorder. Since in the EU significant benefit of an OMP has to be established, we determined for each follow-on OMP for which development was continued on what grounds significant benefit was assumed by the sponsor. Data were collected from the public domain only.

**Results:**

The likelihood of a rare disorder with an approved OMP to obtain at least one follow-on OMP development was strongly associated with disease prevalence, turnover of the first OMP, disease class, disease-specific scientific output and age of onset. Out of a total of 120 follow-on OMPs only one follow-on OMP could be identified for which development was discontinued upon approval of the first OMP for the same rare disorder. Only a substantial level of discontinuation of follow-on OMP development would have indicated the existence of a market monopoly. Moreover, sponsors that continued development of a follow-on OMP predominantly assumed that their product had an improved efficacy compared to the first approved OMP.

**Conclusions:**

This study provides evidence that absence of follow-on OMP development is a matter of time or market size, rather than that the market exclusivity incentive of the European Orphan Drug Regulation creates a market monopoly.

## Background

The current estimate of number of rare disorders is 6,000 to 8,000, many of which are of genetic origin, and affect children at a very early age. In the United States (US), a rare disease is defined as a disease that affects less than 200,000 inhabitants [1]. In the EU, besides affecting fewer than five per 10,000 inhabitants, a rare disease needs to be life-threatening or chronically debilitating [1]. The total number of patients in Europe and the US suffering from a rare disorder is estimated at 55 million, and as such rare disorders are considered a real health issue [2,3]. Because of the rarity, the cost of developing and marketing a medicinal product to diagnose, treat or prevent a rare condition would not be recovered by the expected sales of the medicinal product under normal market conditions. Therefore, in several jurisdictions specific legislation has been introduced to stimulate the development of drugs for rare diseases, so-called orphan drugs [4,5].

Since the European Union (EU) Regulation on Orphan Medicinal Products (OMP) came into force in 2000, more than 60 Orphan Medicinal Products (OMPs) have been approved for marketing and more than 830 medicinal products have received an orphan designation (OD) in the EU within the first decade of this regulation [6,7]. Just like the United States Orphan Drug Act [8-10], the EU Orphan Drug Regulation is highly appreciated for its role in creating a favorable orphan drug development environment [10,11]. However, this apparent success of the legislations has also been questioned. Joppi et al. argued that the large number of designated orphan drugs may provide false hope to patients with a rare disorder, because only a small percentage of these designated orphan drugs have obtained marketing approval in the EU [12,13]. Moreover, both in the EU and US, certain disease classes - in particular oncology - are associated with a high number of orphan designations and approvals [9,14,15]. In brief, the development of orphan drugs is stimulated through a number of regulatory and economic incentives, of which a market exclusivity period of seven and ten years in the US and the EU, respectively, is regarded the foremost one [1,14].

Recently, Roos et al. claimed that the highly praised market exclusivity incentive basically creates a market monopoly that in their view has allowed manufacturers to charge 'exorbitant' prices for orphan drugs [16]. Tambuyzer in contrast argued that "if an approved OMP is currently the only product on the market, it is either because a company was the first to develop a treatment for this disease and competitors have yet to enter the market or because the market is too small to attract competition, rather than because the incentives have created a monopoly" [17]. To our knowledge the hypothesis that the market exclusivity incentive serves as a disincentive for the development and marketing of another OMP, a so-called follow-on OMP, next to the first approved OMP has never been tested before.

Review of the EU Community Register for Orphan Medicinal Products revealed that for several rare disorders with an approved OMP no other OMPs have been approved or are currently under development [6]. However, it also revealed that for some other rare disorders in the EU one or more OMPs have been approved next to the first OMP. For example, sildenafil, sitaxentan, iloprost and ambrisentan have all been approved as treatment for pulmonary arterial hypertension next to bosentan, the first OMP [6]. In the EU it is allowed to approve a follow-on OMP next to the first OMP, provided that significant benefit to those affected by the condition can be established. According to Commission Regulation (EC) No 847/2000 [18], significant benefit means a clinically relevant advantage or a major contribution to patient care, and is generally justified by "demonstration of potentially greater efficacy, an improved safety profile and/or more favorable pharmacokinetic properties than existing methods" [19].

Taking the aforementioned into account it remains unclear whether the market exclusivity incentive serves as a disincentive for follow-on OMP development or not. Therefore, the objective of this study was to determine whether the market exclusivity incentive of the EU Orphan Drug Regulation results in a market monopoly as argued by Roos et al. [16], or that absence of a follow-on OMP for a rare disorder for which already an approved OMP exists in the EU is a matter of time or market size as claimed by Tambuyzer [17]. First, the impact of various market-, product- and disease-related characteristics on follow-on OMP development was determined by comparing rare disorders with an approved OMP and at least one follow-on OMP, with rare disorders with an approved OMP and no follow-on OMP. Next, we determined whether manufacturers continued or discontinued development of a follow-on OMP upon approval of the first OMP for the intended rare disorder. The presence of considerable discontinuation of follow-on OMP development would suggest the existence of a market monopoly. Finally, since in the EU significant benefit of an OMP has to be established, we also determined for each follow-on OMP for which development was continued on what grounds significant benefit was assumed by the sponsor.

## Methods

### Characterization of rare disorders with an approved OMP

The study included all rare disorders that obtained at least one approved OMP for the treatment of that rare disorder in the EU between the start of the EU Regulation on Orphan Medicinal Products (April 2000) and 31 December 2008. The study was limited to rare disorders with an approved treatment, since only one rare disorder with an approved OMP for the diagnosis or prevention of that disorder could be identified. For each rare disorder with an approved OMP the EU register of Orphan Medicinal Products was checked for follow-on OMPs between the start of the EU Regulation on Orphan Medicinal Products (April 2000) and 30 April 2010. The reason for extending the cut-off date was to maximize detection of follow-on OMPs. A follow-on OMP was defined as: (a) another approved OMP for the same rare disorder in Europe (e.g. both agalsidase bèta and agalsidase alfa are approved for the treatment of Fabry disease in Europe) or (b) another medicinal product with a European orphan designation (OD) for the same disorder. Only OMPs that were under development at or after marketing approval of the first OMP for the same rare disorder were identified as follow-on OMP and included in the study. An OMP designated before approval of the first OMP for the same rare disorder, but whose development was discontinued before approval of the first OMP was not considered as follow-on OMP. Finally, rare disorders included in the study were divided into two groups: (a) rare disorders having an approved OMP and at least one follow-on OMP ('Follow-on' group), and (b) rare disorders having an approved OMP, but no follow-on OMP ('no Follow-on' group).

### Factors elucidating follow-on OMP development

Data were collected for each rare disorder on product-, market- and disease-related characteristics to get insight in factors elucidating follow-on OMP development. Selection of data was limited to the public domain and essentially similar to Heemstra et al. [20,21].

Market-related characteristics included data on the prevalence of the rare disorder, the annual turnover of the first OMP, and whether the first OMP was designated or approved outside Europe. Product-related characteristics included data on the pharmaceutical formulation. Disease-related characteristics included data on the disease class, the disease-specific scientific output (i.e. the number of scientific publications) and whether the rare disorder was a childhood, chronic or inheritable disorder [20,21]. A full list of these characteristics, definitions and the (main) public sources used, is depicted in table 1.

**Table 1:** Definitions and sources used for market-, product- and disease-related characteristics of Orphan Medicinal Products (OMPs)

Characteristic	Definitions	(Main) public source
**Market-related characteristics**		
prevalence	< 1 per 100,000 between 1 - 50 per 100,000	Orpha.net (report series, Nov 2009)
annual turnover first OMP	maximum annual turnover of the first OMP	Sponsor documents
first OMP designated outside EU	first OMP obtained an orphan designation	FDA Orphan Drug
	inside and outside Europe	Product database
first OMP authorized outside EU	first OMP obtained marketing authorization	FDA Orphan Drug
	inside and outside Europe	Product database
**Product-related characteristics**		
pharmaceutical formulation	parenteral oral	EPARs
**Disease-related characteristics**		
disease class (ICD-10)	other ICD-10 classes C00-D48 (oncologic disorders)	Orpha.net
disease-specific scientific output	number of scientific publications in the period 1976-2008 in PubMed for a specific rare disorder	PubMed
inheritable disease	majority of cases caused by genetic inheritance	Orpha.net
chronic disease	disease duration is generally over 3 months	Orpha.net
childhood disease	majority of diagnosis before age 18	Orpha.net

The characteristics of rare disorders having at least one follow-on OMP ('Follow-on' group) and rare disorders with no follow-on OMP ('no Follow-on' group) were compared using univariate analyses. Odds ratios (OR) and 95% confidence intervals (CI) were calculated for each characteristic. All analyses were done using SPSS, version 16.0.

### Effect of marketing authorization of first OMP on follow-on OMP development

Upon marketing approval of a first OMP, sponsors of a follow-on OMP for the same rare disorder could either decide to continue or discontinue development. In this study a sponsor continued development if: (a) the orphan designation date of the follow-on OMP, based on the EU orphan drug register, was after the date of marketing authorization of the first OMP or (b) if continuation of development was verified in the public domain for follow-on OMPs having a designation date prior to the approval date of the first OMP. As a result, the following two groups were discerned: (a) sponsors that continued development of a follow-on OMP upon marketing approval of the first OMP and (b) sponsors that discontinued development of a follow-on OMP upon marketing approval of the first OMP.

### Characterization of assumptions of significant benefit of follow-on OMPs

The European Medicines Agency (EMA) guideline COMP/15893/2009 outlines the necessary level of evidence to support assumptions of significant benefit [19]. Significant benefit is justified in the EU by demonstrating an improved efficacy, a better safety or a major contribution to patient care. In this study the concept of significant benefit included these three categories. Data regarding the assumptions of significant benefit were collected using sources in the public domain. Firstly, European Public Assessment Reports (EPARs) were consulted for follow-on OMPs that were successfully developed and approved in the EU. An EPAR provides information regarding the significant benefit of a follow-on OMP compared to earlier approved OMPs for the same rare disorder. Secondly, Summaries of Opinion were consulted provided that the follow-on OMP obtained an orphan designation after approval of the first OMP for the same rare disorder. Finally, sponsor documents (e.g. sponsor website, annual reports, press releases) were consulted to determine the assumptions of significant benefit of follow-on OMPs that obtained an orphan designation before approval of the first OMP for the same rare disorder. All collected assumptions of significant benefit were compared and mapped using central key words of the mentioned EMA guideline [19] for each of the three categories (for example mechanism of action for improved efficacy, side effects for better safety and way of administration for a major contribution to patient care).

## Results

### Characterization of rare disorders with an approved OMP

From the start of the EU Regulation on Orphan Medicinal Products (April 2000) up to 31 December 2008, 58 OMPs intended for the treatment of a rare disorder obtained marketing approval in the EU [6]. Although some products (e.g. imatinib and dasatinib) have been approved for more than one rare disorder, most approved OMPs are intended as treatment for only one specific rare disorder. Collectively, the 58 approved OMPs have been authorized as treatments for 44 different rare disorders. Of these 44 rare disorders, 26 had at least one follow-on OMP ('Follow-on' group) and 18 did not have a follow-on OMP ('no Follow-on' group), as reflected in table 2.

**Table 2:** Rare disorders with an approved OMP in EU until 31 December 2008

Rare disorder (N = 44)	First OMP (INN-name)	Other ODs* (N = 149)
Fabry disease	agalsidase alfa/agalsidase beta**	1
Chronic myeloid leukemia	imatinib	7
Gaucher disease	miglustat	3
Acute promyelocytic leukemia	arsenic trioxide	2
Pulmonary arterial hypertension	bosentan	12
Acromegaly	pegvisomant	2
Gastrointestinal stromal tumors	imatinib	3
Familial adenomatous polypopsis	celecoxib	2
NAGS-deficiency	carglumic acid	-
Treatment prior to hematopoietic progenitor cell		
transplantation	busulfan	-
Mucopolysaccharidosis type I	laronidase	-
Essential thrombocythaemia	anagrelide	-
Wilson's disease	zinc acetate dihydrate	1
High-grade dysplasia in Barrett's oesophagus	porfimer sodium	-
Patent ductus arteriosus	ibuprofen	-
Adrenal cortical carcinoma	mitotane	1
Hairy cell leukemia	cladribine	1
Narcolepsy-cataplexy	sodium oxybate	1
Chronic pain requiring intraspinal analgesia	ziconotide	-
Tyrosinaemia type I (hereditary)	nitisinone	-
Dermatofibrosarcoma protuberans	imatinib	-
Acute lymphoblastic leukemia	clofarabine	16
Anthracycline extravasations	dexrazoxane	-
Renal cell carcinoma	sorafenib	18
Mucopolysaccharidosis type VI	galsulfase	-
Glycogen storage disease type II	alglucosidase alpha	1
Chronic iron overload requiring chelation therapy	deferasirox	2
Hypereosinophilic syndrome	imatinib	1
Myelodysplastic syndromes	imatinib	7
Hepatocellular carcinoma	sorafenib	9
Soft tissue sarcoma	trabectedin	7
Paroxysmal nocturnal haemoglobinuria	eculizumab	-
Sickle cell syndrome	hydroxycarbamide	3
Multiple myeloma	lenalidomide	14
Lennox-Gastaut syndrome	rufinamide	-
Severe primary IGF-1 deficiency	mecasermin	-
Mucopolysaccharidosis type II	idursulfase	-
Severe myoclonic epilepsy in infancy (Dravet's syndrome)	stiripentol	-
Homocystinuria	betaine anhydrous	-
Systemic sclerosis	bosentan	3
Chronic myelomonocytic leukemia	azacitidine	-
Hyperphenylalaninaemia	sapropterin dihydrochloride	2
Angioedema (hereditary)	icatibant	3
Acute myeloid leukemia	histamine dihydrochloride	27

* Other ODs in the EU under development for treatment of the specific rare disorder between the start of the EU Orphan Drug Regulation (April 2000) and 30 April 2010.

** Both agalsidase alfa and agalsidase beta obtained market approval at the 3th of August 2001 in the EU.

A review of the EU Community Register of Orphan Medicinal Products revealed that the 26 rare disorders in the 'Follow-on' group encompass a total of 163 orphan designations (ODs) between the start of the EU Orphan Drug Regulation (April 2000) and 30 April 2010 in the EU [6]. Analysis of the 163 ODs showed that development of 13 orphan drugs had been discontinued before marketing approval of the first OMP for the same rare disorder. In addition, one orphan drug was intended for the diagnosis rather than for the treatment of a rare disorder. From the remaining 149 ODs, 120 orphan drugs were identified as being under development at or after approval of the first OMP and included as follow-on OMP in this study. Despite an in depth search into multiple sources in the public domain, for the remaining 29 ODs we were unable to determine whether these orphan drugs were still under development at or after approval of the first OMP for the same rare disorder. Consequently, these ODs could not be identified as follow-on OMPs and were not included in the study.

### Factors elucidating follow-on OMP development

The results of the univariate comparisons between rare disorders with an approved OMP and at least one follow-on OMP (n = 26) and rare disorders with an approved OMP and no follow-on OMP (n = 18) are shown in table 3. For some of the characteristics statistically significant relations were observed, however, the association was strongest for the scientific output of a specific disorder. A rare disorder with more than 450 scientific publications in PubMed in the period 1976-2008 was associated with a 25-fold increase in the chance to obtain at least one follow-on OMP compared to a rare disorder with less than 450 scientific publications in this period (OR = 25.0; CI = 2.8-226.1). Furthermore, the results showed that oncologic disorders (ICD-10 class C00-D48) with an approved OMP have an 8-fold increased likelihood to obtain at least one follow-on OMP compared to rare disorders in other ICD-10 classes (OR = 8.0; CI = 1.5-42.0). A statistically significant relation was also found for the annual turnover of the first approved OMP. Rare disorders having a first approved OMP with more than 50 million US dollar annual sales have an almost 5-fold higher probability to obtain a follow-on OMP than rare disorders having a first approved OMP with annual sales below 50 million US dollar (OR = 4.8; CI = 1.1-21.4). Finally, a striking result is that rare disorders with an age of onset in childhood have a 5-fold lower chance to obtain at least one follow-on OMP than rare disorders with an age of onset in adulthood (OR = 0.2; CI = 0.1-0.8).

**Table 3:** Comparison between rare disorders with follow-on and no follow-on OMPs: Descriptive statistics and univariate analyses

Characteristic	Indicator	Rare disorders			
		Total N = 44	'Follow-on' Group N = 26	'No Follow-on' Group N = 18	**Odds Ratio (95% Confidence Interval**)
**Market-related**					
prevalence	< 1 per 100,000	9	0*	9	reference level
	between 1 - 50 per 100,000	34	26	8	not applicable
turnover first	< 50 mil. US $	11	4	7	reference level
OMP	> 50 mil. US $	26	19	7	4.8 (1.1-21.4)
designated outside	no	8	5	3	reference level
EU	yes	36	21	15	0.8 (0.2-4.1)
approved outside	no	11	7	4	reference level
EU	yes	33	19	14	0.8 (0.2-3.2)
**Product-related**					
pharmaceutical	parenteral	20	9	11	reference level
formulation	oral	24	17	7	3.0 (0.9-10.3)
**Disease-related**					
disease class	other ICD-10 classes	29	13	16	reference level
	C00-D48	15	13	2	8.0 (1.5-42.0)
disease-specific	< 450 publications	10	1	9	reference level
scientific output	> 450 publications	34	25	9	25.0 (2.8-226.1)
childhood	adulthood	22	17	5	reference level
	childhood	17	7	10	0.2 (0.1-0.8)
chronic	non-chronic	5	3	2	reference level
	chronic	34	19	15	0.8 (0.1-5.7)
inheritable	non-inheritable	28	18	10	reference level
	inheritable	16	8	8	0.6 (0.2-1.9)

* Zero cell count/no follow-on OMP

The descriptive statistics showed a strong association between the disease prevalence and the likelihood to obtain at least one follow-on OMP. All rare disorders with a first approved OMP and at least one follow-on OMP had a prevalence between 1/100,000 and 50/100,000, whereas rare disorders with a first approved OMP and no follow-on OMP were more or less equally divided over both prevalence categories (< 1/100,000 and 1-50/100,000).

### Effect of marketing authorization of first OMP on follow-on OMP development

To elucidate whether the instrument of market exclusivity served as disincentive, we determined the effect of marketing authorization of a first OMP on follow-on development. Overall, our results show that development of follow-on OMPs is continued after marketing approval of a first OMP for the same rare disorder. Out of a total of 120 follow-on OMPs only one follow-on OMP, PI-88 of the Australian company Progen, could be identified for which development was discontinued. According to the company the decision of discontinuation "was made based on a number of factors, but was primarily driven by the competitive pressures in Europe and North America of Bayer's launching a Nexavar^® ^trial amongst the same patients" [22].

### Characterization of assumptions of significant benefit of follow-on OMPs

Assumptions of significant benefit were found for 106 of the 119 follow-on OMPs in the EU. For the remaining 13 follow-on OMPs no assumptions of significant benefit could be identified, predominantly due to lack of information in sponsor documents for follow-on OMPs that obtained an orphan designation (OD) before marketing approval of the first OMP (n = 10). For the other three follow-on OMPs the Summary of Opinion did not provide clear information regarding the assumption of significant benefit (e.g. "the product might be of potential significant benefit"). Consequently, these follow-on OMPs were not included in the study.

For several follow-on OMPs (n = 22) more than one assumption of significant benefit was found, resulting in a total of 130 different assumptions, as depicted in table 4. Most assumptions of significant benefit (85.5%; n = 111) were related to the concept of improved efficacy. Within this category, most sponsors assumed that their follow-on OMP would be of significant benefit due to a different mechanism of action, followed by treatment of a subgroup of patients. The assumptions of significant benefit for the remaining 19 follow-on OMPs were related to an improved safety profile (n = 9) or a major contribution to patient care (n = 10) in comparison with the first OMP for the same rare disorder, respectively. More than half of the follow-on OMPs in the category of improved safety were assumed to have fewer side effects compared to existing treatments. With regard to the contribution to patient care it was observed that, except for one follow-on OMP, all assumptions were related to the mode of administration.

**Table 4:** Assumptions of significant benefit of the follow-on OMPs for which development was continued

Category of significant benefit	Classes of significant benefit	Assumptions	
		N = 130	%
**Improved efficacy**	Different mechanism of action	47	36.2
	Sub-group (including patients who do not respond		
	on current treatments)	27	20.8
	Alternative/additional treatment	14	10.8
	Improve the long-term outcome of the patient	12	9.2
	More effective (unspecified)	10	7.7
	Improve the treatment (unspecified)	1	0.8
	**Total**	**111**	**85.5**
**Improved safety**	Fewer side effects	5	3.8
	Increased tolerability	2	1.5
	Improved safety (unspecified)	2	1.5
	**Total**	**9**	**6.8**
**Contribution to patient care**	Mode of administration	9	6.9
	Wider availability	1	0.8
	**Total**	**10**	**7.7**

## Discussion

In the interest of patients with a rare disorder, but also of other stakeholders, better understanding of the effect of the market exclusivity incentive on follow-on OMP development for a rare disorder for which an approved OMP exists is warranted. The objective of this study was to determine whether the market exclusivity incentive of the EU Orphan Drug Regulation results in a market monopoly, as argued by Roos et al. [16], or that absence of follow-on OMPs is a matter of time or market size, as claimed by Tambuyzer [17]. First, the impact of various market-, product- and disease-related characteristics on follow-on OMP development in the EU was determined. Comparison of rare disorders with a first approved OMP and at least one follow-on OMP ('Follow-on' group) with rare disorders with a first approved OMP and no follow-on OMP ('no Follow-on group') revealed disease prevalence, turnover of the first OMP, disease class, disease-specific scientific output and age of onset as predictors of follow-on OMP development. Although it was not possible to perform a multivariate analysis and to test whether any of the aforementioned characteristics were mutually related, it is suspected that some level of mutual relationship may exist. Heemstra et al. showed that scientific output for oncologic rare disorders in general is higher than for other rare disorders, which suggests some degree of mutual relationship between disease class and scientific output [20]. Also, the prevalence of a rare disorder and turnover of the first OMP may to some extent be mutually related. Despite the possibility of mutual relationships, our findings are in line with several studies that focused on characteristics that play a driving role in the orphan drug development process [20,23-25].

The lower likelihood on follow-on OMP development for rare childhood disorders is most likely a natural consequence of the general reluctance of the pharmaceutical industry to invest in the development of specific therapies for a population that is small in number and fragmented into several age groups with often unique requirements [26,27]. To stimulate paediatric drug development in the EU Regulation (EC) No 1901/2006 on medicinal products for paediatric use came into force in January 2007 [28]. Through a number of regulatory and economic incentives, including extension of the market exclusivity period from ten to twelve years for OMPs intended for a paediatric indication, the legislation intends to have a positive impact on paediatric drug development, although risks and pitfalls remain [29]. Conducting clinical trials for rare indications, especially very rare ones, remains difficult due to a number of well-known practical limitations (e.g. lack of proper diagnosis, finding enough patients, geographical distribution of patient population) [30]. Successful drug development requires thorough disease knowledge, such as its etiology and pathophysiology [25]. The latter explains our finding that, next to initiation of an orphan drug development program [20], the level of disease-specific scientific output increases the likelihood on follow-on OMP development. It also explains our observation that for rare oncologic diseases the likelihood on follow-on OMP development is higher than for other ICD disease classes. Considerable public and private expenditure on research and a high-level of transnational research infrastructure have resulted in oncology having the highest scientific output in the area of rare diseases [31,32]. Consequently, oncology represents an attractive indication for the pharmaceutical sector [33,34], resulting in the highest number of first and follow-on OMPs in development as well as approved [6]. Because of its dynamic and divergent nature, biomedical research can result in several competing hypotheses on the possible causes of diseases, and several promising targets and therapeutical approaches [35]. This makes biomedical research an extremely complex process to manage, and as a result translation of disease knowledge into (follow-on) OMP development requires time.

In contrast to the claim by Roos et al. that the market exclusivity incentive basically creates a market monopoly that in their view allows manufacturers to charge 'exorbitant' prices for orphan drugs [16], we found that turnover of the first OMP in fact increases the likelihood on follow-on OMP development. The presence of an approved OMP for a rare disorder is a clear indication that development and marketing of a therapy for that specific rare disorder is attainable. However, the mere presence of an approved OMP for a rare disorder is not sufficient; a certain level of profitability is apparently an important prerequisite for the initiation or continuation of the development of a follow-on OMP.

Our finding that prevalence of a rare disease is associated with an increased likelihood on follow-on OMP development provides corroborative evidence for an element in Tambuyzer's hypothesis that if an approved OMP is currently the only product on the market, this can be explained because the market is too small to attract competition [17]. Acemoglu and Linn more generally revealed that an increase in the potential market size for a drug category correlates with an increase in the number of new drugs in that category [24]. The importance of prevalence of a rare disease, which can be regarded as a surrogate marker for market size, was already confirmed in other stages of the orphan drug development process. Heemstra et al. showed that prevalence is a predictor for the translation of rare disorder research into the initiation of an orphan drug development program [20]. Yin reported that "the US Orphan Drug Act has led to a significant and sustained increase in new trials" among more prevalent rare diseases, but not for less prevalent rare diseases [23].

Tambuyzer's hypothesis is further supported by our finding that in the EU all manufacturers of follow-on OMPs upon approval of the first OMP for the same rare disorder decided to continue development of their product, except for one. Only a substantial level of discontinuation of follow-on OMP development would have indicated the existence of a market monopoly. Discontinuation of several follow-on OMPs was found, however, reasons for discontinuation were related to conducting clinical trials, focus on another more promising indication, registration failure and lack of funding. Apparently, discontinuation of follow-on OMPs is not directly associated with market approval of a first OMP for the same rare disorder, but rather due to safety-, efficacy-, or economic-related factors similar to the majority of regular drug development discontinuation [36]. Interestingly, we found that 15 of the 120 follow-on OMPs (12.5%) subsequently obtained a marketing authorization in the EU between the start of the legislation and April 2010. This is considerably higher than the 45 of 528 orphan designations (8.5%) that were approved in the EU between the start of the legislation and 2007 [12]. One explanation for this observation could be that the majority of follow-on OMPs is in development by large pharmaceutical firms (data not shown), whereas most orphan designated products are being developed by Small and Medium-sized Enterprises (SMEs) [37]. In general, SMEs have less experience in (orphan) drug development, which was reported by Heemstra et al. as an important predictor for OMP approval [21].

Finally, we found that sponsors that continue development of a follow-on OMP in the EU predominantly assume that their product has an improved efficacy compared to the first OMP. Establishing the assumption of significant benefit based on an improved safety and/or major contribution to patient care may be either more difficult for or less attractive to manufacturers of follow-on OMPs. The EMA guideline on significant benefit mentions that "a theoretical risk with an authorized product cannot be compared with a theoretical lack of risk with the product under development" [19]. However, most applications for an orphan designation by a sponsor are done in the late preclinical or early clinical testing stage, which doesn't include extensive human safety testing. Moreover, serious adverse events with authorized orphan medicinal products have been limited until now [38]; only sitaxentan was voluntarily withdrawn from the market by Pfizer in 2010 due to new information on two cases of fatal liver injury [39]. The reason for the limited number of follow-on OMPs with significant benefit based on an assumption of a major contribution to patient care may be best explained by a current lack of interest by the pharmaceutical industry. Increased patient involvement and demand may result in shifting focus to novel innovative drug delivery systems that will allow more selective, more precise, less frequent dosing of the orphan medicinal product [40,41].

## Conclusion

This study provides evidence for the hypothesis that approval of the first OMP for a rare disorder, and consequently a market exclusivity period of ten years, does not serve as a disincentive for the development and marketing of a follow-on OMP for the same indication in the EU. We found that absence of follow-on OMP development is more a matter of time or market size as argued by Tambuyzer [17], rather than the creation of a market monopoly as claimed by Roos et al. [16]. Apart from market size, turnover of the first OMP, disease class, disease-specific scientific output and age of onset were identified as additional predictors of follow-on OMP development. In general, regardless of approval of the first OMP for the intended rare disorder, development of a follow-on OMP is continued and primarily based on the assumption of improved efficacy.

Finally, within the area of rare diseases the role of patient organizations is strong, a real added value, and continuously growing [40,41]. It will be interesting to monitor if this increased patient involvement and demand will result in expanding the focus of the pharmaceutical industry to novel innovative drug delivery systems that will allow more selective, more precise, less frequent dosing of the orphan medicinal product [40]. Ultimately, we believe that, next to improved efficacy, this kind of follow-on OMP development truly represents an important improvement in current care of patients with a rare disorder.

## Competing interests

The authors declare that they have no competing interests.

## Authors' contributions

RDV and SVW were responsible for the study idea. AB designed and performed the study, was the primary responsible person for data collection and data evaluation. RDV, EM and SVW participated in the study design and data evaluation. RDV and EM were involved in the supervision of this study. AB wrote together with RDV the manuscript and SVW and EM revised the manuscript. All authors read and approved the final manuscript.
